# Clinical utility of FDG PET/CT for primary and recurrent papillary renal cell carcinoma

**DOI:** 10.1186/s40644-021-00393-8

**Published:** 2021-02-25

**Authors:** Guozhu Hou, Dachun Zhao, Yuanyuan Jiang, Zhaohui Zhu, Li Huo, Fang Li, Wuying Cheng

**Affiliations:** 1 Department of Nuclear Medicine, Peking Union Medical College Hospital Chinese Academy of Medical Sciences and Peking Union Medical College, Beijing, 100730 China; 2 Beijing Key Laboratory of Molecular Targeted Diagnosis and Therapy in Nuclear Medicine, Beijing, 100730 China; 3 Department of Pathology, Peking Union Medical College Hospital Chinese Academy of Medical Sciences and Peking Union Medical College, Beijing, 100730 China

**Keywords:** Papillary, Renal cell carcinoma, FDG, PET/CT

## Abstract

**Purpose:**

Papillary renal cell carcinoma (RCC) is the second most common subtype of RCC, after clear cell RCC. This study aimed to investigate the usefulness of FDG PET/CT in primary and recurrent papillary RCC, and the role of staging FDG PET/CT in predicting survival.

**Methods:**

A total of 66 patients with histopathologically confirmed papillary RCC who underwent either staging or restaging FDG PET/CT scans (30 had staging scans only, 28 had restaging scans only, 8 had both) were retrospectively included in this study. The sensitivity and specificity of restaging FDG PET/CT for detecting recurrence were assessed by histopathology and/or clinical follow-up as standard reference.

**Results:**

Staging FDG PET/CT scans were performed in 38 patients, of which 31 (81.5%) showed FDG-positive primary renal lesions. The SUVmax of high-grade (WHO grade 3 and 4) papillary RCCs were significantly higher than that of low-grade (WHO grade 1 and 2) tumors (9.44 ± 6.18 vs 4.83 ± 3.19, *P* = 0.008). The SUVmax was not significantly different between type 1 and type 2 papillary RCCs (5.71 ± 2.88 vs. 6.99 ± 5.57, *P* = 0.563). Of the 38 patients, 12 developed disease progression during the follow-up period. Patients with primary tumor SUVmax> 5.85 were associated with significantly shorter progression-free survival (PFS) than those with tumor SUVmax≤5.85 (*P* = 0.005). Restaging FDG PET/CT scans were performed in 36 patients with suspected recurrent papillary RCCs. FDG PET/CT showed a sensitivity and specificity of 100 and 72.7% for detecting recurrent disease. Comparison of PET/CT scans with CT/MRI imaging was available in 23 patients. FDG PET/CT revealed additional findings in 11 patients, mainly including lymph node and bone metastases. FDG PET/CT findings led to change in management in 5.3% (2/38) of patients in the staging setting and 16.7 (6/36) of patients in the restaging setting.

**Conclusions:**

FDG PET/CT had a sensitivity of 81.5% for detecting primary papillary RCC, and tumor SUVmax derived from staging FDG PET/CT was a predictor of PFS. In the restaging process of papillary RCC, FDG PET/CT was very effective for detecting recurrent disease.

**Supplementary Information:**

The online version contains supplementary material available at 10.1186/s40644-021-00393-8.

## Introduction

Renal cell carcinoma (RCC) accounts for approximately 90% of renal malignancies and is a heterogenous group of various subtypes of cancer [1, 2]. Papillary renal cell carcinoma (papillary RCC) is the second most common variant of RCC following clear cell renal cell carcinoma (clear cell RCC) and represents about 10–15% of all RCCs [3]. Based on the pathological findings, papillary RCCs are further divided into two subtypes (type 1 and type 2) that are histologically distinct. Compared to type 1 tumors, type 2 papillary RCCs are considered more aggressive with poorer prognosis [4]*.* Surgery is currently the treatment of choice for organ-confined tumor, while locally advanced or metastatic disease often requires pharmacological or targeted therapy [5]. It is generally believed that compared to clear cell RCC, papillary RCCs are associated with a more favorable prognosis, including higher survival rates and lower incidence of metastasis [6, 7]. Imaging-based characterization of papillary RCC using computed tomography (CT) and magnetic resonance imaging (MRI) have been addressed in many studies [8–12].

^18^F-FDG PET/CT, a noninvasive molecular imaging modality, has been extensively applied in clinical practice. It has emerged as one of the most important imaging methods for staging, restaging, and monitoring therapeutic response in most malignancies. Unlike for most other malignancies, the application of FDG PET/CT in the preoperative evaluation of RCC is limited, primarily due to the physiological excretion of FDG from the kidneys [13]. Although FDG PET/CT shows unfavorable results in the detection and characterization of primary renal lesions, the metabolic parameters derived from FDG PET/CT plays an important role in the prediction of patient’s prognosis [14]. FDG PET/CT is also more effective in the detection of recurrent RCC, and thus may affect therapeutic strategies [15, 16]. However, to date, studies on the use of FDG PET/CT in RCC predominantly enrolled patients with clear cell RCC [17–21], and only a few studies included a small proportion of papillary RCC [16, 22, 23]. Research of FDG PET/CT focusing on papillary RCC is scarce and limited to case reports [24–26]. This retrospective study aimed, 1) to assess the usefulness of FDG PET/CT in the evaluation of primary and suspected recurrent papillary RCC, 2) to evaluate the prognostic value of staging FDG PET/CT in papillary RCC patients.

## Methods

### Patients

We retrospectively reviewed patients with histopathologically confirmed papillary RCC who received either staging FDG PET/CT or restaging FDG PET/CT for suspected recurrence in our hospital from January 2010 to July 2020. For the staging group, inclusion criteria were as follows: newly diagnosed, histologically confirmed pRCC; nephrectomy or biopsy performed at our hospital; FDG PET/CT performed before initial treatment; interval between PET/CT scan and surgery/biopsy within 30 days; availability of patient’s follow-up information. Exclusion criteria were as follows: therapy prior to FDG PET/CT; nephrectomy or biopsy not performed at our hospital; incomplete clinical records. For the restaging group, inclusion criteria were as follows: histologically proven pRCC; history of previous treatment for primary pRCC; FDG PET/CT performed for restaging; the presence of standard reference (histopathology or clinical/imaging follow-up for at least 6 months after PET/CT scan. Exclusion criteria were as follows: FDG PET/CT performed during the first 4 weeks of surgery; lack of definite reference standard. Finally, 66 patients met the inclusion criteria and were included in this study. Sixty-three patients received surgery to remove the primary renal tumor. For the remaining 3 patients, the histopathological diagnosis of papillary RCC was based on the biopsy of metastatic lesion or primary renal lesion. A total of 30 patients underwent only staging PET/CT scans, 28 patients had only restaging PET/CT scans, and 8 patients underwent both staging and restaging PET/CT scans. The patients comprised of 46 men and 20 women with a median age of 53 years (range, 21–84; mean, 52.8 ± 16.6). Nuclear grading and histopathological subtypes (type 1 and 2 papillary RCC) data were available in 34 patients who underwent staging FDG PET/CT. Nuclear grading was determined based on World Health Organization (WHO)/International Society of Urological Pathology (ISUP) grading system [3]. This retrospective study was approved by the institutional review board, and patient consent was not required.

### FDG PET/CT study

All patients fasted for at least 4 to 6 h and had a blood glucose level of less than 200 mg/dL, before receiving an intravenous administration of ^18^F-FDG (5.5 MBq/kg). After FDG injection, they rested for approximately 60 min. PET/CT images were acquired supine from the skull base to the midthigh level using a combined PET/CT biograph (Siemens Company, Germany). A low-dose CT scan was obtained for attenuation correction and anatomical reference. PET scans were acquired in a three-dimensional mode (2–3 min/bed position).

### Image analysis

All images were retrospectively read by 2 experienced nuclear medicine physicians. Semiquantitative analysis of abnormal FDG uptake for lesions was performed using the maximum standardized uptake value (SUVmax). The SUVmax of primary renal tumor was measured by drawing a region of interest over the primary tumor avoiding physiological activity of the renal calyces as much as possible. Staging FDG PET/CT scan was defined as positive if the tumor uptake was higher than normal renal parenchyma or surrounding extrarenal soft tissues in the case of exophytic tumor, while was considered negative if the tumor uptake was lower than /equal to normal renal parenchyma or surrounding extrarenal soft tissue. The tumor size was defined as the longest diameter of the primary renal lesion. Restaging PET/CT scan was defined as positive if at least one site of abnormal uptake suspected of recurrence was detected. Specifically, a lesion was positive if the FDG activity was moderately or markedly increased relative to surrounding soft tissues or comparable normal structures. On the contrary, a lesion showing no or faint uptake of FDG was regarded as negative even if a suspicious recurrent lesion had been detected by CT. True positive PET/CT scan corresponded to an abnormal finding confirmed by histopathology or follow-up. PET/CT scan was deemed false positive if the histopathological results from a suspected FDG-positive lesion revealed no evidence of malignancy or the lesion remitted/showed no change without therapy during the follow-up period. A negative PET/CT scan was considered false negative if the lesion was detected by CT and was confirmed by histopathology or by clinical or imaging progression. True negative PET/CT scan corresponded to the absence of abnormal imaging findings confirmed by negative histopathological results or lack of recurrence during the follow-up.

### Reference standard

The lesions found on staging FDG PET/CT images were verified by histopathology from surgery or biopsy. For both ethical and practical reasons, not every suspected recurrent lesion observed on restaging FDG PET/CT could be investigated by histopathology. Therefore, for restaging FDG PET/CT scan, a reference standard was determined on the basis of histopathology (if available) and clinical/imaging follow-up (CT, ultrasonography, PET/CT, MRI) for at least 6 months. For example, lesions that showed an increase in number/size on CT or MR or an increase in FDG uptake on follow-up PET/CT, or showed a response to anticancer therapy during the follow-up were considered as recurrent lesions even if a histopathological examination was unavailable.

### Statistical analysis

SUVmax was compared between high-grade and low-grade papillary RCCs, as well as between type 1 and type 2 tumors using the Mann-Whitney U test. Prognostic value of SUVmax derived from staging FDG PET/CT in papillary RCC patients was investigated. Progression-free survival (PFS) was defined as the time from primary surgery to the appearance of clinical or radiological progression. To determine the prognostic factors associated with PFS, univariate analysis of variables, including tumor SUVmax, primary tumor size, age, gender, pTNM stage, nuclear grade was performed. Receiver operating characteristic (ROC) curve analysis was used to determine the optimal cutoff values for SUVmax, primary tumor size and age. Patients were then divided into two groups according to the cutoff values for SUVmax, primary tumor size and age. Progression-free survival rates were obtained using the Kaplan-Meier method. Variables with log-rank *P* < 0.05 were selected for multivariate analyses by means of a cox regression model. To evaluate the usefulness of FDG PET/CT in the restaging of papillary RCC, sensitivity, specificity, positive predictive value (PPV), and negative predictive value (NPV), and accuracy were calculated on a per-patient basis. *P* value less than 0.05 was considered statistically significant. The statistical analyses were performed using SPSS (IBM SPSS Statistics for Windows, Version 21.0. Armonk, NY).

## Results

### Staging FDG PET/CT in primary papillary RCC

#### Primary staging

**Fig. 1:**
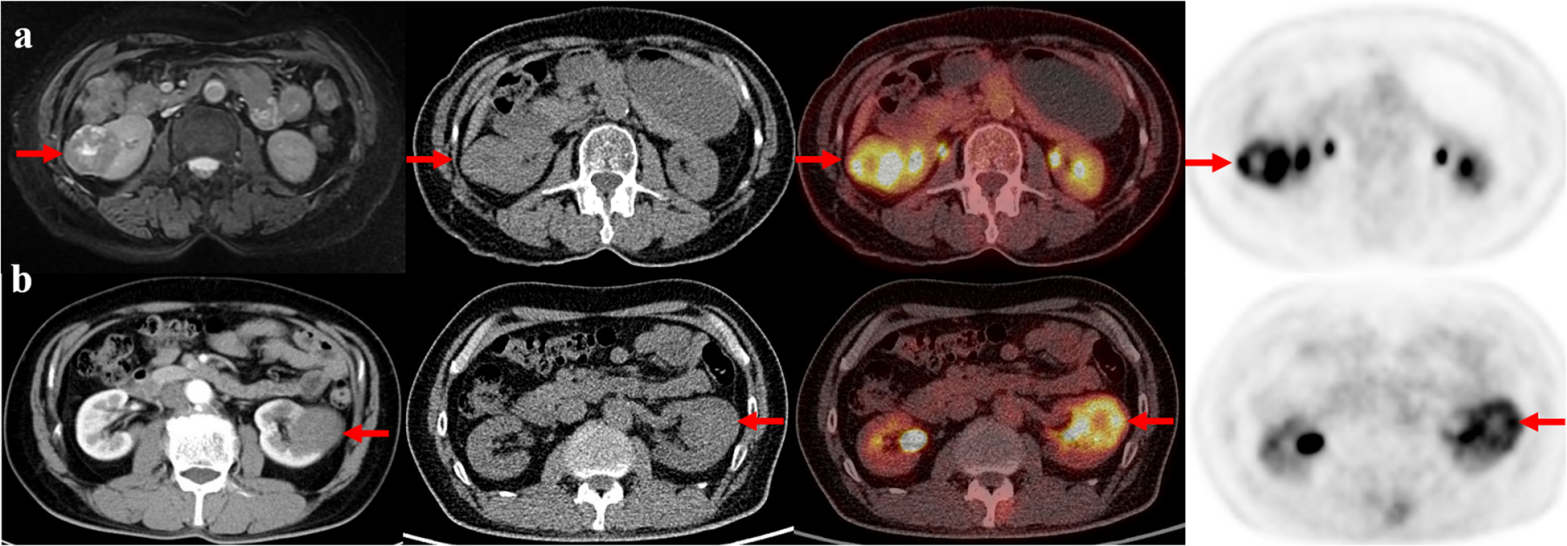
Representative cases. **a** The axial images from left-to-right are MR, coregistered CT, PET/CT fusion and PET. A 63-year-old woman with type 2 papillary renal cell carcinoma (WHO G3) in the right kidney (arrows). The primary tumor size is 4.4 cm, and the tumor SUVmax is 10.4. **b** The axial images from left-to-right are diagnostic contrast-enhanced CT, coregistered CT, PET/CT fusion and PET. A 61-year-old man with type 1 papillary renal cell carcinoma (WHO G2) in the left kidney (arrows). The primary tumor size is 4.2 cm, and the tumor SUVmax is 6

**Fig. 2:**
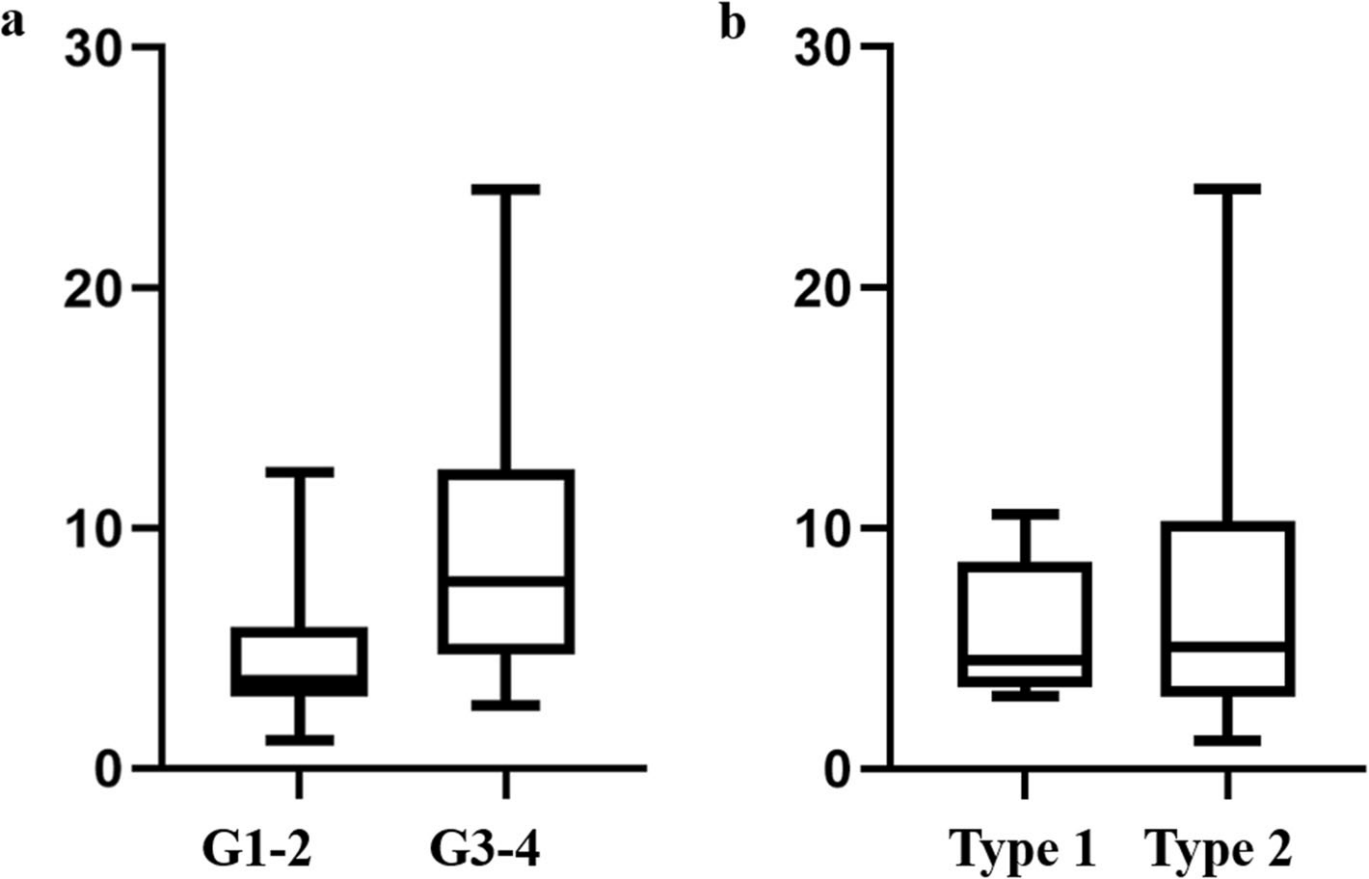
Box plots showed that the SUVmax was significantly higher in high-grade tumors than in low-grade tumors (*P* = 0.008, **a**), while the SUVmax was not significantly different between type 1 and type 2 tumors (*P* = 0.563, **b**)

Primary staging scans were performed in 38 patients (Table 1). Thirty-one patients showed FDG-positive renal lesions on PET/CT, resulting a sensitivity of 81.5% (31/38) (Fig. 1). The SUVmax of primary renal lesions ranged from 1.2 to 24.1 (median, 4.8; mean, 6.88 ± 5.27). The SUVmax of high-grade (G3 and G4) papillary RCCs were significantly higher than that of low-grade (G1 and G2) tumors (9.44 ± 6.18 vs 4.83 ± 3.19, *P* = 0.008). While the SUVmax was not significantly different between type 1 and type 2 papillary RCCs (5.71 ± 2.88 vs. 6.99 ± 5.57, *P* = 0.563) (Fig. 2). The size of primary renal lesions ranged from 0.9 to 15.6 cm (median, 4.0 cm; mean, 5.04 ± 3.45).

**Table 1:** Characteristics of patients who underwent pretherapeutic FDG PET/CT

Characteristic	Value
Number of patients	38^a^
Age, years	
Median	56
Range	24–82
Gender, men/women	25/13
Follow-up duration, months	
Median	32
Range	4–115
Primary tumor size (cm)	
Median	4
range	0.9–15.6
pTNM stage	
I/II	32
III/IV	6
Nuclear grade (WHO grade)	
G1-G2	20
G3-G4	14
Histological subtype	
Type 1	7
Type 2	27
Treatments	
Radical nephrectomy	19
Partial nephrectomy	18
Targeted therapy	1
Disease progression	12
Histology	
Papillary RCC	38

^a^Eight of the 38 patients also underwent restaging FDG PET/CT scans

Of the 38 patients, management was changed in 2 patients (5.3%) as a direct result of the PET/CT findings. In one patient, FDG PET/CT identified unsuspected left supraclavicular and mediastinal nodal metastasis, which was biopsied and confirmed as metastatic papillary RCC. The treatment plan was then switched from surgery to targeted therapy. In the other case, management was changed as a result of detection of synchronous lung adenocarcinoma on FDG PET/CT. In the remaining 36 patients, 4 patients were found with metastasis at the time of diagnosis, including regional nodal metastases (*n* = 3), adrenal gland metastasis (*n* = 1), and renal venous tumor thrombus (*n* = 2).

#### Survival analysis

Among the 38 patients who underwent staging FDG PET/CT examinations, 12 developed recurrence of papillary RCC during the follow-up period. Of note is that the patient with synchronous lung adenocarcinoma at the time of diagnosis did not show evidence of recurrence of either RCC or lung adenocarcinoma during the follow-up period (53 months). The median PFS time was 32 months (range, 4–115 months; mean, 42.36 ± 33.86 months). From the ROC curve analyses, the optimal cutoff value of SUVmax, primary tumor size, and age for estimating PFS were 5.85, 4.3 cm and 48.5 years, respectively.

### FDG PET/CT in suspected recurrent papillary RCC

#### Diagnostic performance

**Fig. 3:**
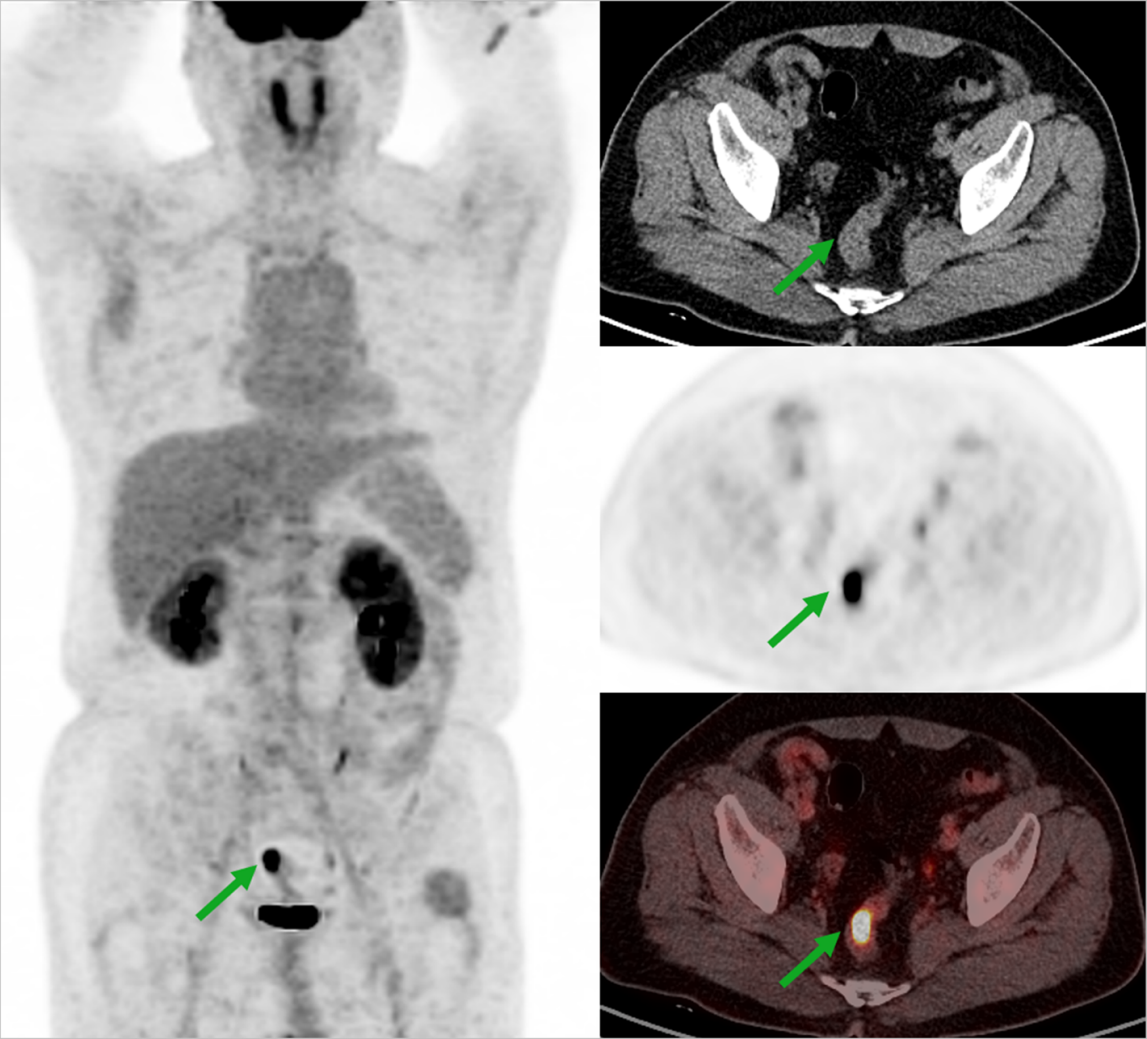
A 52-year-old man had right partial nephrectomy for papillary renal cell carcinoma. Six months after surgery, surveillance FDG PET/CT showed a focal intense uptake in the sigmoid colon (SUVmax, 14.7; red arrows), which was confirmed as benign adenoma on biopsy. There was also incidental finding of physiologic uptake by muscle in the left hip and right shoulder. The patient did not develop recurrence during the follow-up period (84 months)

Restaging PET/CT scans for suspected recurrent papillary RCC were performed in 36 patients (Table 2). FDG PET/CT showed a positive finding in 28 patients and a negative finding in 8 patients. To confirm the presence of recurrent disease, the results of FDG PET/CT was compared with histopathology in 12 patients, and with clinical and/or diagnostic imaging evaluation in 24 patients. According to the reference standard, FDG PET/CT had true positive findings in 25 patients, and true negative findings in 8. No patient showed false negative finding in this study. In 3 patients, FDG PET/CT showed false positive findings. One was due to the benign adenoma in the sigmoid colon (Fig. 3). For the other 2 patients, the false positive findings were related to the postoperative scars. Thus, the patient-based sensitivity, specificity, PPV, and NPV, and accuracy of FDG PET/CT were 100, 72.7, 89.2, 100, and 91.6%, respectively.

**Table 2:** Characteristics of patients who underwent restaging FDG PET/CT

Characteristic	Value
Number of patients	36^a^
Age, years	
Median	53
Range	21–84
Gender, men/women	25/11
Duration after primary treatments, months	
Median	14
Range	1–121
Primary treatments	
Radical nephrectomy	18
Partial nephrectomy	16
Targeted therapy	2
Histology	
Papillary RCC	36

^a^Eight of the 36 patients also underwent pretherapeutic FDG PET/CT scans

#### Correlation with anatomic imaging

**Fig. 4:**
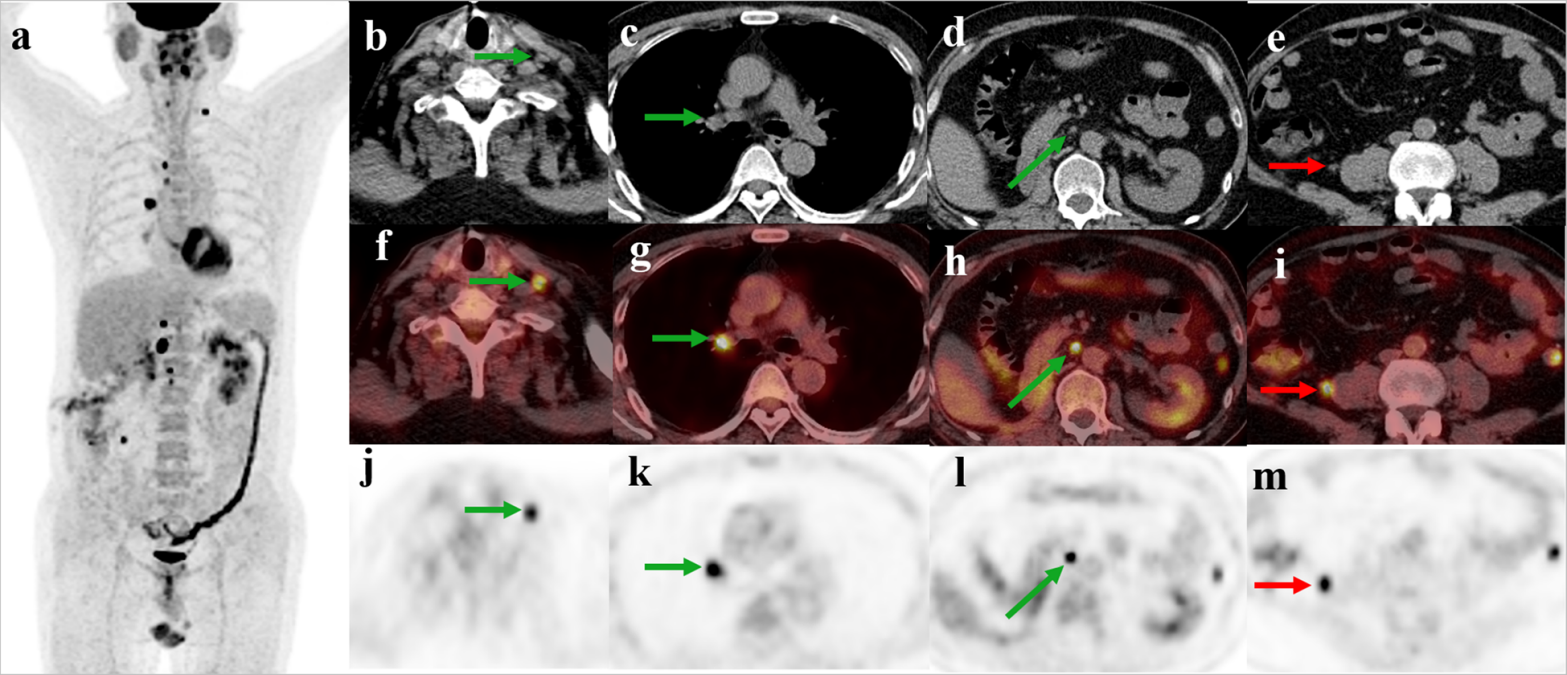
A 53-year-old man presented with multiple lymph node recurrences 3 months after right radical nephrectomy for renal cell carcinoma. Unenhanced diagnostic CT revealed enlarged lymph node in the retroperitoneum. FDG PET/CT (**a**-**m**) showed additional lymph node metastases (green arrows) in the left supraclavicular fossa, mediastinum, right hilum, retroperitoneum, and a hypermetabolic soft-tissue nodule (bule arrows) near the right psoas muscle, which were not large enough to be interpreted as positive at CT scan. Symmetric parotid gland uptake and post-surgical related uptake in the left renal region were also seen on PET/CT

As described in Table 3, the most frequent site for recurrence was in the lymph node, followed by the bone. Correlation between FDG PET/CT and conventional imaging was available for 23 patients. The PET/CT and conventional imaging findings were concordant in 12 patients. PET/CT detected additional findings in the remaining 11 patients (11/23, 47.8%) (Fig. 4), including lymph node metastases in 7 patients, bone lesions in 4, subcutaneous/intramuscular metastases in 4, peritoneal dissemination in 2, adrenal gland metastasis in 1.

**Table 3:** Sites of recurrence on FDG PET/CT

Sites of recurrence	Number of patients
Lymph node	16
Bone	10
Soft tissue	9
Peritoneum	3
Local recurrence	2
Liver	2
Adrenal gland	2
Mesentery	1
Lung	1

Based on the findings on FDG PET/CT, change in management was undertaken in 6 of 36 patients (16.7%) in the restaging setting. One patient being treated with targeted therapy alone was subsequently treated with additional radiotherapy after FDG PET/CT detected bone metastases. In 4 patients, management was changed from observation to targeted therapy after FDG PET/CT detected multiple metastases. In the remaining one patient, an intramuscular soft tissue metastasis was detected by FDG PET/CT, which was treated with surgery.

## Discussion

In the present study, we found that the tumor SUVmax derived from staging FDG PET/CT could predict prognosis in patients with papillary RCCs. The application of FDG PET/CT in oncology is based on the correlation between tumor glucose metabolism and the degree of aggressiveness, and metabolic quantitation by SUVmax may play a role in the prediction of patient’s prognosis. Nakajima et al. evaluated the prognostic role of preoperative FDG PET/CT in patients with primary RCC, including 115 clear cell RCC, 15 papillary RCC, and 9 chromophobe RCC. They found that higher tumor SUVmax (> 3.83) was significantly associated with decreased PFS time in patients with RCC [22]. Our results also demonstrated that patients with higher tumor SUVmax (> 5.85) had a significantly shorter PFS time than those with lower SUVmax (≤5.85), suggesting that SUVmax may serve as a prognostic factor in patients with papillary RCCs. Lower tumor FDG uptake might reflect a less aggressive biomolecular status of renal carcinoma, which is associated with better prognosis.

For the evaluation of primary RCC, the role of FDG PET/CT is considered quite limited. A review article by Wang et al. noted that FDG PET or PET/CT had a sensitivity of only 62% for the detection of primary RCC [27]. In a recent study, Nakanishi et al. assessed the diagnostic performance of 11C-choline PET/CT and FDG PET/CT in RCC, and reported a sensitivity of 86.7 and 53.3% for primary renal tumors, respectively [20]. This low sensitivity rate is explainable. Previous studies investigating the diagnostic performance of FDG PET/CT for primary RCC generally included only clear cell RCC or predominantly clear cell RCC, and low-grade clear cell RCC made up a substantial proportion of this entity. As several studies characterizing the FDG uptake of RCC reported, low-grade clear cell RCC had significantly lower FDG activity than high-grade clear cell RCC, which was mainly responsible for the low sensitivity for RCC detection.

In the present study, where only papillary RCCs were included, FDG PET/CT had a relatively favorable performance for primary renal tumor detection with a sensitivity of 81.5%. The mean tumor SUVmax of papillary RCC in our study was 6.88 ± 5.27. Our results also showed that high-grade papillary RCCs had significantly higher SUVmax than that of low-grade tumors. Similarly, in the study of Nakajima et al., which included 17 papillary RCC cases, the mean SUVmax of high-grade tumors was significantly greater than in low-grade tumors [23]. In their study, the 17 papillary RCCs comprised of 16 type 2 cases and a single type 1 case, and the comparison of SUVmax between type 1 and type 2 tumors was not performed. In this study, histological subtype data was available in 34 patients who underwent staging FDG PET/CT, including 27 type 2 and 7 type 1 cases. The mean SUVmax of type 2 tumors was only slightly higher than that of type 1 tumors (5.71 ± 2.88 vs. 6.99 ± 5.57), and the difference was not statistically significant (*P* = 0.563). Our result suggested that FDG PET/CT may not be able to distinguish papillary RCC subtypes. Still, the number of type 1 cases was quite small in this study. Future studies with larger sample size are needed to validate this result.

Although the utility of FDG PET/CT in assessing primary renal tumor is controversial, it has been considered more effective in the detection of metastatic or recurrent disease, thus affecting therapeutic management. Previous studies have reported various sensitivity and specificity rates of FDG PET/CT in utilization for recurrent RCC [28, 29]. In an earlier study by Majhail et al., FDG PET/CT was reported to have a low sensitivity (63.6%) for detecting distant metastasis in a population of 24 clear cell RCC patients [30]. This reported low sensitivity probably delayed the use of FDG PET/CT in RCC. Kumar et al. evaluated 103 scans from 63 patients with suspected recurrent RCC, FDG PET/CT exhibited 90% sensitivity and 91% specificity in the study [31]. The largest reported study included 315 patients who underwent FDG PET/CT for recurrent RCC, a sensitivity of 100% and specificity of 100% was observed [28]. Nakatani et al. investigated FDG PET/CT scans of 23 patients suspected of recurrent RCC, including 19 clear cell RCCs and 4 papillary RCCs. They reported an overall sensitivity of 81%. Of note was that all 4 false-negative cases in their study were clear cell RCC, thus resulting 100% sensitivity for papillary RCC and 75% sensitivity for clear cell RCC [32]. Different from previous studies, our study included only patients with the second most common type of renal cancer, papillary RCC. And our analysis showed a higher sensitivity rate (100%) than most of the previously published studies for detecting recurrence and no false-negative case weas observed in our series.

Our study also showed relevant results regarding the clinical management of pRCC patients. FDG PET/CT could detect occult metastasis that may be undetected by conventional imaging or distant metastasis that could not be assessed on available imaging due to limited/regional field of view, thus influencing therapeutic management. In the staging setting, therapeutic management was modified in 5.3% of patients. FDG PET/CT detected distant lymph node metastasis in one patient, thus rendering the patient unsuitable for curative surgery and avoiding the possible morbidity and mortality associated with surgery. In the other patient, management was changed due to the detection of synchronous primary malignancy. In this study, FDG PET/CT findings also led to management changes in 16.7% of patients in the restaging setting. One patient being treated with targeted therapy received additional radiotherapy for bone metastases detected by FDG PET/CT. In 4 patients, management was switched from observation to targeted therapy following the detection of multiple metastases on PET/CT. In the remaining one patient, the detection of intramuscular soft tissue metastasis by FDG PET/CT led to surgery. To date, few studies have assessed the impact of FDG PET/CT in the management of patients affected by RCC. In a study on 104 patients with recurrent RCCs, Alongi et al. reported that FDG PET/CT altered management in 43% cases [16]. The rate of changes in management in the current study was lower than that of Alongi et al., which might be due to the facts that the number of patients with recurrent RCCs in our study is much smaller than theirs and that our study included only pRCCs patients while the study of Alongi et al. included predominantly clear cell RCCs patients.

Several limitations of this study should be pointed out. First, this is a retrospective study that uses existing data, the study could be subject to selection bias. Second, the number of type 1 papillary RCC cases was quite small compared to that of type 2 cases. Thus, the analysis of comparison of SUVmax between histological subtypes would be under-powered. Third, histopathology would have been an ideal reference standard, but was not feasible in all patients and for each FDG-avid lesion because of practical and ethical reasons. Additional limitation of this study was the small number of enrolled patients.

## Conclusions

FDG PET/CT had a sensitivity of 81.5% for detecting primary papillary RCC, and tumor SUVmax derived from staging FDG PET/CT was a predictor of PFS. In the restaging process of papillary RCC, FDG PET/CT was very effective for detecting recurrent disease.

## Supplementary Information

**Additional file 1.**

**Additional file 2.** Kaplan-Meier survival graphs showing significant differences in progression-free survival between the groups categorized according to pTNM stage, SUVmax, primary tumor size, and WHO grade. High pTNM stage, SUVmax > 5.85, primary tumor size > 4.3, high WHO grade (G3 and G4) were associated with decreased progression-fee survival

## Data Availability

Not applicable.

## Abbreviations

FDG: Fluorodeoxyglucose
PET: Positron emission tomography
CT: Computed tomography
SUVmax: Maximum standardized uptake value
RCC: Renal cell carcinoma
PFS: Progression-free survival
WHO: World Health Organization
ISUP: International Society of Urological Pathology
ROC: Receiver operating characteristic
PPV: Positive predictive value
NPV: Negative predictive value
