# Supplementary material for: Clinical utility of FDG PET/CT for primary and recurrent papillary renal cell carcinoma
Source: Cancer Imaging. 2021 Feb 25;21:25. doi: 10.1186/s40644-021-00393-8 (PMC7908760; doi:10.1186/s40644-021-00393-8)
Supplement: Supplementary file 1 — Additional file 1. [file 40644_2021_393_MOESM1_ESM.docx]

*Survival analysis*

In the univariate analyses for PFS, pTNM stage, SUVmax, primary tumor size, and nuclear grade (WHO grade) were significantly associated with decreased PFS (P < 0.05 each) (Fig 3). In the multivariate analysis using the Cox regression method, only nuclear grade was found to be an independent prognostic factor of PFS (*P* = 0.033; hazard ratio, 10.137; 95% CI, 1.205–85.270).
