# Supplementary figures and images for: Clinical utility of FDG PET/CT for primary and recurrent papillary renal cell carcinoma
Source: Cancer Imaging. 2021 Feb 25;21:25. doi: 10.1186/s40644-021-00393-8 (PMC7908760; doi:10.1186/s40644-021-00393-8)

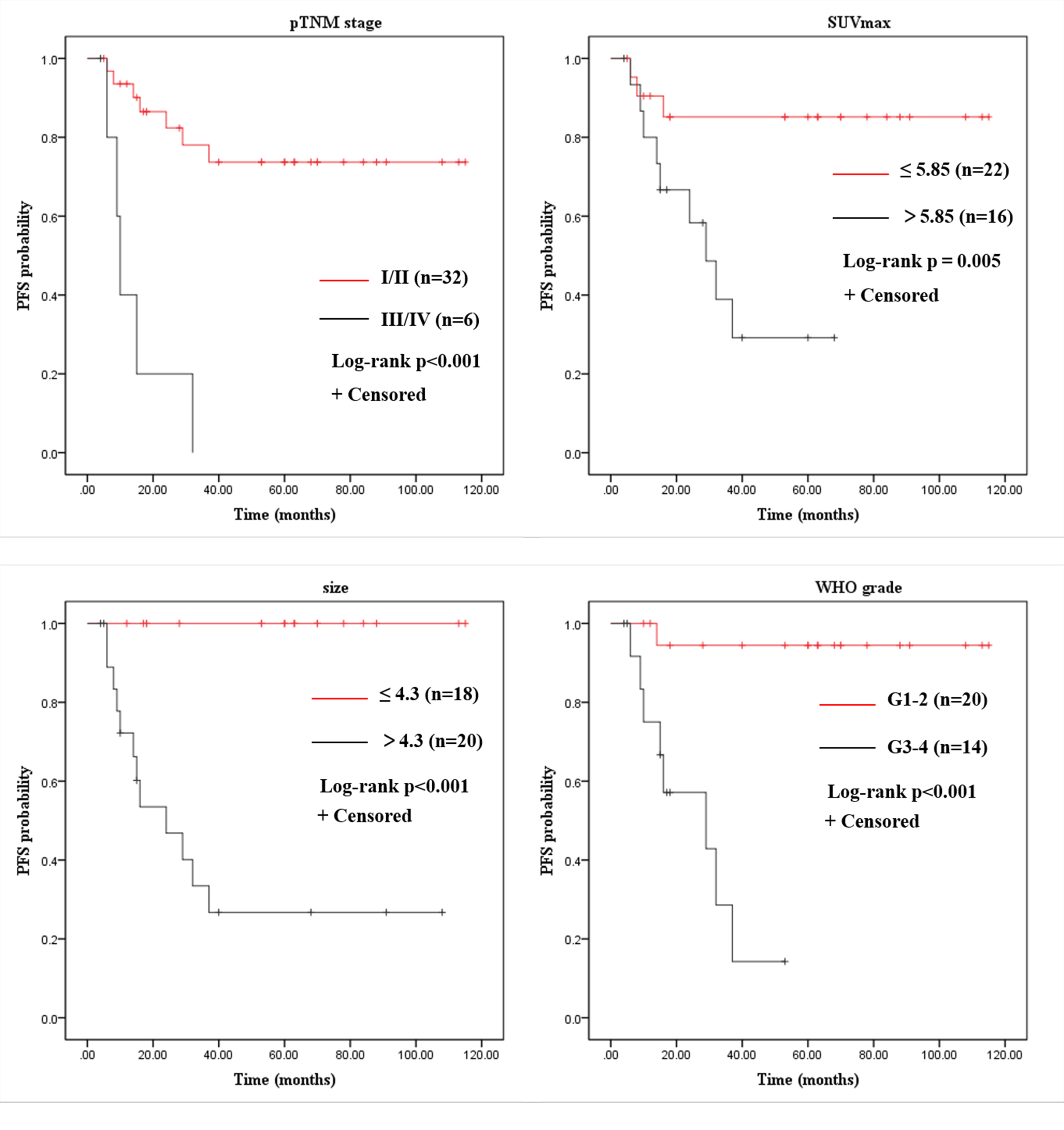

Supplement: Supplementary file 2 — Additional file 2. Kaplan-Meier survival graphs showing significant differences in progression-free survival between the groups categorized according to pTNM stage, SUVmax, primary tumor size, and WHO grade. High pTNM stage, SUVmax > 5.85, primary tumor size > 4.3, high WHO grade (G3 and G4) were associated with decreased progression-fee survival [file 40644_2021_393_MOESM2_ESM.tif]
